# Surgical Management of the Cesarean Scar Ectopic Pregnancy: A Case Report

**DOI:** 10.1155/2013/525187

**Published:** 2013-12-22

**Authors:** Anisodowleh Nankali, Mina Ataee, Haleh Shahlazadeh, Sara Daeichin

**Affiliations:** ^1^Obs & Gyn Department, Maternity Research Center, Imam Reza Hospital, Parastar Boulevard, Sorkheh Lyjeh, Kermanshah University of Medical Sciences, Kermanshah 67188-17757, Iran; ^2^Obs & Gyn Department, Imam Reza Hospital, Parastar Boulevard, Sorkheh Lyjeh, Kermanshah University of Medical Sciences, Kermanshah 67188-17757, Iran

## Abstract

Cesarean scar pregnancy is one of the rarest forms of ectopic pregnancy. Little is known about its incidence and natural history. The diagnosis and treatment of cesarean scar pregnancy (CSP) is challenging. The authors reported here a case of cesarean scar pregnancy (CSP) with hypovolemic shock that underwent emergency laparotomy with resection of ectopic mass. The patient was discharged from the hospital without any complications.

## 1. Introduction

Cesarean scar pregnancy (CSP) is an ectopic pregnancy implanted in the myometrium at the site of a previous cesarean section scar [1].

It is a rare and life-threatening condition [2]. However, its incidence is increasing over the years due to the rise in cesarean section rate worldwide [3, 4].

A recent case series estimates an incidence of 1 : 2226 of all pregnancies with a rate of 0.15% in women with a previous cesarean section and a rate of 6.1% of all ectopic pregnancies in women who had at least one case cesarean delivery [5].

Its genesis involves implantation into the myometrium via a microscopic tract or sometimes a dehiscence in the previous uterine scar [6].

Several types of conservative treatment have been used such as dilatation and curettage, excision of trophoblastic tissues (laparotomy or laparoscopy) [7, 8], local and/or systemic administration of methotrexate [9], bilateral hypogastric artery ligation associated with trophoblastic evacuation, and selective uterine artery embolization combined with curettage and/or MTX administration [10, 11].

Laparotomy followed by wedge resection of the lesion (hysterotomy) should be considered in women who do not respond to conservative medical and/or surgical treatments or present too late [12, 13].

Some consider this as the best treatment option [2].

In this paper we describe a case of viable cesarean scar pregnancy that was presented with hypovolemic shock and successfully treated via hysterotomy and evacuation of pregnancy.

## 2. The Case

A 25-year-old female, and gravid 2 para 1, with a previous history of cesarean section 5 months ago, was admitted to Imam Reza University Teaching Hospital for lower abdominal pain at 11-weak gestation based on first day of last menstrual period. Abdominal pain started one week ago with exacerbation of pain 3 days before admission. She had mild vaginal bleeding, nausea, and vomiting on the day of admission.

Physical examination demonstrated distention of abdomen. The patient blood pressure was 90/60 mmHg, her pulse rate was 139/beat/min, her respiratory rate was 16/Min and body temperature of her was = 37.6°C.

Generalized abdominal tenderness was noted upon palpation; speculum examination revealed slight bleeding through cervical oss. In bimanual examination the uterus seemed to be of 12 weeks gestation.

**Figure 1:**
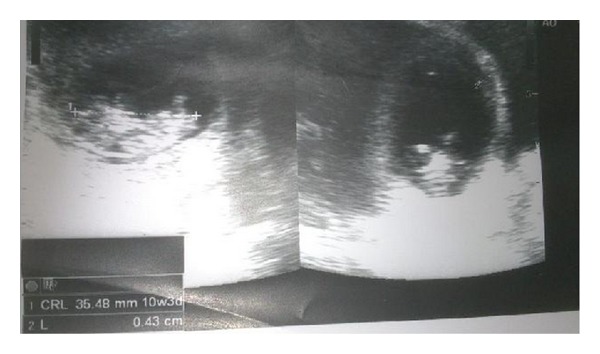
Transabdominal ultrasound showing gestational sac with fetus in the lower uterine segment.

Transabdominal sonography revealed a gestation sac with a live 10/5-week gestation fetus and a fetal cardiac activity in the anterior wall of lower body of uterus in the region of the previous cesarean scar; myometrial thickness surrounding was less than 10 mm Figure 1.

Relatively too much hemorrhagic fluid was in cul-de-sac and paracolic area.

Sonographic findings suggested cesarean scar pregnancy. She was resuscitated with fluids. Her hemoglobin level was 8/9 g/dL, Hct: 28/3%, Plt: 234 × 1000 mm3, PT: 13.6 sec, Activity: 76.3, INR: 1.3, PTT: 36 sec, Renal and liver function tests were normal.

**Figure 2:**
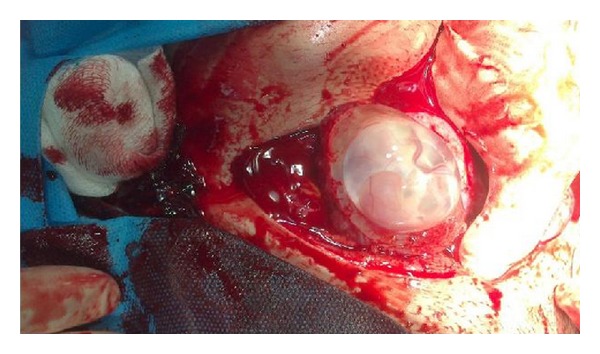
Intact gerstational sac along with placental tissue seen protruding through previous cesarean sacr defect.

Possibility of ruptured scar ectopic pregnancy was kept and exploratory laparotomy was performed. Intraoperatively we found 1.5 litters of hemoperitoneum with ruptured uterine scar through which amniotic sac with a live fetus was protruding; see Figure 2.

**Figure 3:**
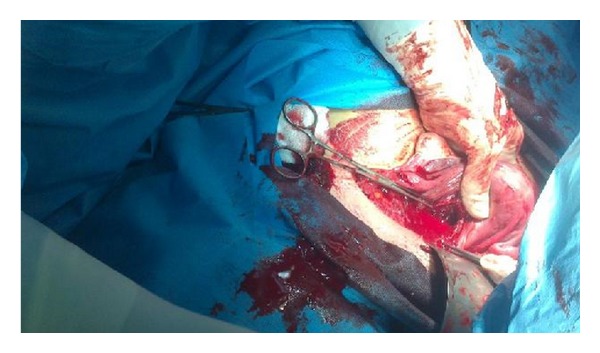
Uterus was evacuated and uterine defect repaired in two layers.

Uterus was evacuated and uterine defect repaired in two layers; see Figure 3. The patient received two units of blood intraoperatively.

Her postoperative period was uneventful and she was discharged on the 4th postoperative day.

## 3. Discussion

Cesarean scar pregnancy is the rarest kind of ectopic pregnancy, but because of the increasing number of cesarean deliveries its incidence has been rising to be about 1/2000 normal pregnancy [14].

Cesarean scar pregnancy rate accounts for 6% of ectopic pregnancies among women with a prior cesarean delivery [14, 15]. The incidence does not appear to correlate with the number of cesarean deliveries.

The mechanism for implantation in this location is believed to be migration of the embryo through either the wedge defect in the lower uterine segment or a microscopic fistula within the scar [5, 13, 16].

Adenomyosis, in vitro fertilization, previous dilation and curettage, and manual removal of placenta are risk factors [13, 15, 16].

The clinical presentation ranges from vaginal bleeding with or without pain to uterine rupture and hypovolemic shock [6, 13, 17].

Most of the cases that have been reported were diagnosed early in the first trimester [14].

The diagnosis is made by sonographically visualizing an enlarged hysterotomy scar with an embedded mass [18, 19].

Differential diagnosis includes cervical ectopic pregnancy and placenta accreta [19].

Gestational age at diagnosis ranged from 5 + 0 to 12 + 4 weeks [20].

The present case was admitted at 11 weeks of gestation.

The time interval from the last cesarean section to the diagnosis of cesarean scar pregnancy ranged from 6 months to 12 years.

In the case presented here this time interval was 5 months. This is interesting regarding the shortest interval time that has been reported till now.

Because of the risk of uterine rupture and uncontrollable bleeding, hysterectomy is indicated; however, several types of conservative treatment have been used such as dilation curettage and excision of trophoblastic tissues using laparotomy or laparoscopy [7, 8].

Local and/or systemic MTX administration [10]. Bilateral hypogastric artery ligation, associated with dilation and evacuation under laparoscopic guidance [21]. and selective UAE in combination with curettage and/or MTX injections [10, 21].

The immediate complications of cesarean scar pregnancy are uterine rupture, severe bleeding, need for hysterectomy, and maternal morbidity. Our patient underwent emergency laparotomy and evacuation of product of conception from hysterotomy scar and repair of uterus with traditional methods. She left the hospital with an uneventful postoperative period.

## 4. Conclusion

The ectopic pregnancy within the scar of a previous cesarean delivery can lead to uterine rupture and life-threatening intraperitoneal hemorrhage during the first trimester of pregnancy.

Though a rare event, the incidence of cesarean scar pregnancy seems to be on the rise. An obstetrician is likely to encounter this entity in his or her lifetime.

In women with a history of cesarean scar pregnancy early ultrasound should be performed in subsequence pregnancies in order to establish the location of implantation.
